# The End of the Prince of Wales' Tour

**Published:** 1920-10-16

**Authors:** 


### The End of the Prince of Wales' Tour.

On Monday last H.R.H. the Prince of Wales, returning from his tour of the British Dominions, was enthusiastically welcomed by the population of London, who apart from the previous popularity of the Prince, recognise the large asset he is of Empire and the successful Ambassador he has proved himself amongst friendly nations as well as to our fellow-subjects in distant regions. No section of the public understands the value of the Heir to the Throne more than do those engaged in hospital work, with which His Royal Highness has largely identified himself during recent years. It is well within our memory that the Prince made a successful appeal at Festival Dinners on behalf of the Middlesex Hospital and of the Great Northern Central Hospital, and on May 12, 1919, he took up the Presidency of Guy's Hospital. To show that the holding of the office of President of a great hospital is no nominal thing, on February 5 last the Prince visited Guy's and presided at a special meeting of the General Court of Governors, inaugurating on that occasion the work of an Appeal Committee, and making an exhaustive tour of inspection of the hospital. Following upon the holding of the office by his father and his grandfather, the Prince succeeded to the post of President of King Edward's Hospital Fund for London and presided at the last meeting of the General Council on December 16, 1919, when he read a letter of congratulation to the Fund from His Majesty the King. At the King's Fund meeting the Prince alluded to the fact that the Fund in the first year, when founded by King Edward in 1897, distributed £57,000, and under his father's Presidency the distribution reached £150,000, while in December last it had risen still further to £230,000. We are confident that under the Presidency of the Prince of Wales the helping capacity of King Edward's Fund will still further increase; and hospitals generally, while passing through this difficult and anxious period, have high hopes that the well-known interest taken by the Prince in the work will be an example to all members of the community in bearing a share of the cost and responsibilities of this national service.

